# Activationless Charge Transfer Drives Photocurrent Generation in Organic Photovoltaic Blends Independent of Energetic Offset

**DOI:** 10.1021/jacs.4c11114

**Published:** 2024-11-27

**Authors:** Yifan Dong, Rui Zheng, Deping Qian, Tack Ho Lee, Helen L. Bristow, Pabitra Shakya Tuladhar, Hyojung Cha, James R. Durrant

**Affiliations:** † Department of Chemistry and Centre for Processable Electronics, Imperial College London, London W12 0BZ, United Kingdom; ‡ Straits Institute of Flexible Electronics (SIFE, Future Technologies), Fujian Normal University, Fuzhou, Fujian 350117, China; § Department of Chemistry Education, Graduate Department of Chemical Materials, Institute for Plastic Information and Energy Materials, Sustainable Utilization of Photovoltaic Energy Research Center, Pusan National University, Busan 46241, Republic of Korea; ∥ Department of Hydrogen and Renewable Energy, Kyungpook National University, Daegu 41566, Republic of Korea; ⊥ SPECIFIC and Department of Materials Science and Engineering, Swansea University, Swansea SA1 8EN, United Kingdom

## Abstract

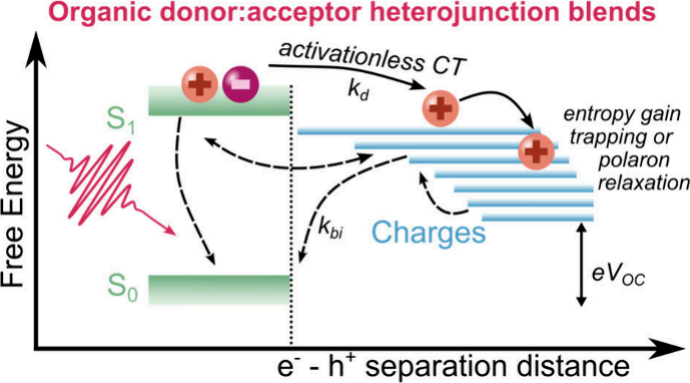

Organic photovoltaics (OPVs) have recently shown substantial progress in enhancing device efficiency, driven in particular by advances in the design of nonfullerene acceptors and the reduction of the energy offset driving exciton separation at the donor/acceptor interface. Herein, we employ temperature-dependent transient absorption spectroscopy to investigate the activation energy for charge generation and recombination in a range of bulk heterojunction blends with nonfullerene acceptors. Remarkably, we find that in all cases charge generation is almost activationless, in the range of 11–21 meV, independent of energetic offset. Geminate recombination is also observed to be almost activationless, with only the kinetics of bimolecular charge recombination being strongly temperature-dependent, with an activation energy >400 meV. Our observation of essentially activationless charge generation, independent of energy offset, strongly indicates that charge generation in such blends does not follow Marcus theory but can rather be considered an adiabatic process associated with the motion of thermally unrelaxed carriers.

## Introduction

Photoexcitation in organic semiconducting materials generates Coulombically bound electron–hole pairs, often referred to as excitons. In bulk-heterojunction (BHJ) organic photovoltaics (OPVs), these excitons are separated by charge transfer at the interface between electron donor (D) and acceptor (A) species.^1−3^ This exciton separation is driven by the electronic energy offset at this interface, often quantified by the difference in energy between the singlet exciton S_1_ of the lower gap component, and resultant charge transfer (CT) states, Δ*E*_S1-CT_. The mechanism of this charge transfer and its dependence on Δ*E*_S1-CT_, as well as other factors such as molecular structure and nanomorphology, remain the subject of widespread study.^4−7^ Such studies have been recently motivated by advances in the development of polymer:nonfullerene acceptor (NFA) blends capable of achieving efficient charge generation even with relatively small energetic offsets, a key factor behind the recent, striking advances in OPV device efficiency.

A key consideration for understanding charge transfer in OPVs is whether this process is thermally activated. Marcus theory, often used to describe charge transfer at such interfaces,^8−13^ predicts that the activation barrier to charge transfer depends on its energetic driving force Δ*E*_S1-CT_. Notably, photosynthesis has evolved reaction centers with energy offsets optimized to enable activationless charge separation, optimizing the kinetics of excited state separation into charges.^14^ Alternatively, other studies of OPV blends have proposed adiabatic mechanisms based on ballistic or hot charge transfer^15,16^ and/or have focused on the activation energy required to separated interfacial “charge transfer” states into free charges.^17^ A further consideration is the potential, for small Δ*E*_S1-CT_, of hybridization/thermal equilibrium between S_1_ and CT states.^18−21^ In the high efficiency PM6:Y6 blend, Perdigón-Toro et al. have reported that charge photogeneration is barrierless despite a low energetic offset,^22^ suggesting this activationless behavior may be a key factor enabling this blend’s high device performance. This conclusion has been supported by Natsuda et al.,^23^ although we note that Ma et al. have proposed a thermally activated pathway for CT state dissociation in this blend.^24^ Other studies have reported slower CT rates in other polymer:NFA blends, on the order of tens of picoseconds, suggested to be associated with small energetic offsets resulting in energetic barriers to charge transfer.^25−27^ Further, it is unclear whether the activationless behavior reported by Perdigón-Toro et al. is unique to PM6:Y6 or more general for organic BHJs and how the activation barrier depends on Δ*E*_S1-CT_. Elucidating this impact is not only of interest to determine the fundamental reaction mechanism but also central to enabling high efficiency OPV devices with minimal energetic losses.

## Results and Discussion

In this study, we employed temperature-dependent transient absorption spectroscopy (TAS) over a picosecond to nanosecond time scale to investigate charge generation and recombination pathways in a series of BHJ blend films. Temperature-dependent TAS measurements allow us to quantify the activation energy (*E*_a_) barriers for charge transfer and recombination. These studies were undertaken on six OPV blend films with a range of driving forces for charge separation, including both fullerene acceptors and NFAs. The study includes blends with varying proportions of geminate versus nongeminate recombination and study of both electron and hole transfer. Remarkably, we find almost activationless charge generation for all the blends studied (*E*_a_ = 11–21 meV), suggesting that this barrierless behavior is not unique to high performance blends such as PM6:Y6 but rather is a more general property of organic BHJ films. The implications of this observation for the charge transfer mechanism and for the design of high performance OPV blends are discussed.

**Figure 1:**
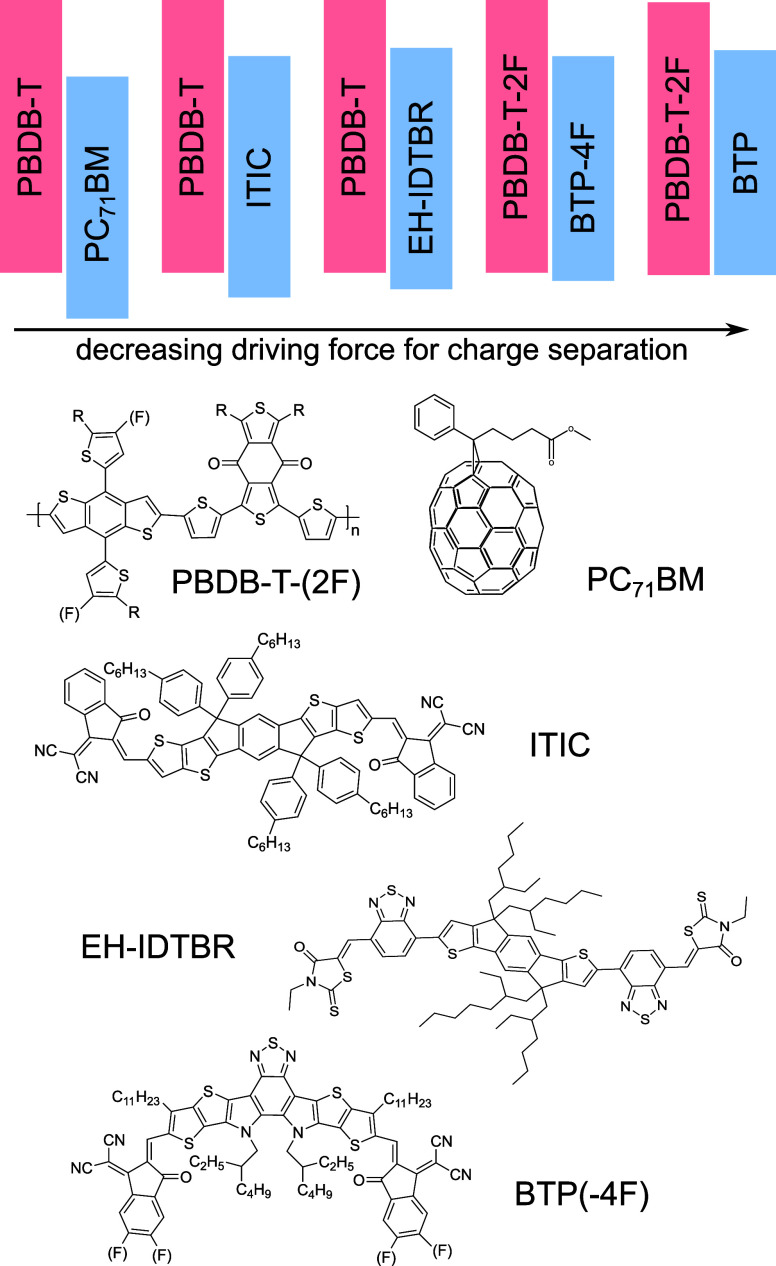
Chemical structures and approximate energetic alignments of the donors and acceptors employed in the BHJ films studied in this work.

Figure 1 shows five of the OPV blend systems investigated in this work, along with their chemical structures (see Figure S1 for device performance). The electron donor materials consist of the archetypal polymer PBDB-T (i.e., PCE12) and its fluorinated derivative PBDB-T-2F (i.e., PM6).^28,29^ In particular, the fluorination of PBDB-T enables the modulation of the energy offset between D and A while maintaining similar morphology and crystallinity, resulting in various driving forces for charge separation in the studied blend systems. The donor PBDB-T was blended with PC_71_BM, ITIC, and EH-IDTBR, while PBDB-T-2F was blended with BTP-4F (i.e., Y6) and BTP. These five blend systems follow a descending order in terms of energy loss *E*_g_ – *V*_OC_ (where *E*_g_ is the optical gap and *V*_OC_ is the open circuit voltage), as detailed below and also illustrated by the trend in indicative HOMO level offsets in Figure 1. We use *E*_g_ – *V*_OC_ as a convenient approximate proxy for the energetic offset driving charge separation Δ*E*_S1-CT_ due to the difficulty in measuring CT energies in small energy offset blends. The significant quenching of photoluminescence (PL) efficiencies, exceeding 80% upon blending, indicates that these blend systems all exhibit relatively efficient exciton dissociation.^30,31^ In addition to these five blend systems, we also investigated a further blend with even lower driving force, PTO2:BTP-4F (as reported in our recent paper^21^), which will be discussed later.

To gain insight into excited state dynamics, we performed ultrafast TAS characterization on these blend films at temperatures from 77 to 330 K. In all cases, we employed selective excitation of the lowest bandgap material and monitored the corresponding electron/hole transfer and subsequent recombination processes over the picosecond to nanosecond time scale. TA spectra were measured in both the visible (450–800 nm) and near-infrared (850–1500 nm) regions under low excitation intensities to minimize nonlinear effects from exciton–exciton annihilation (see Notes S1 and S3).

**Figure 2:**
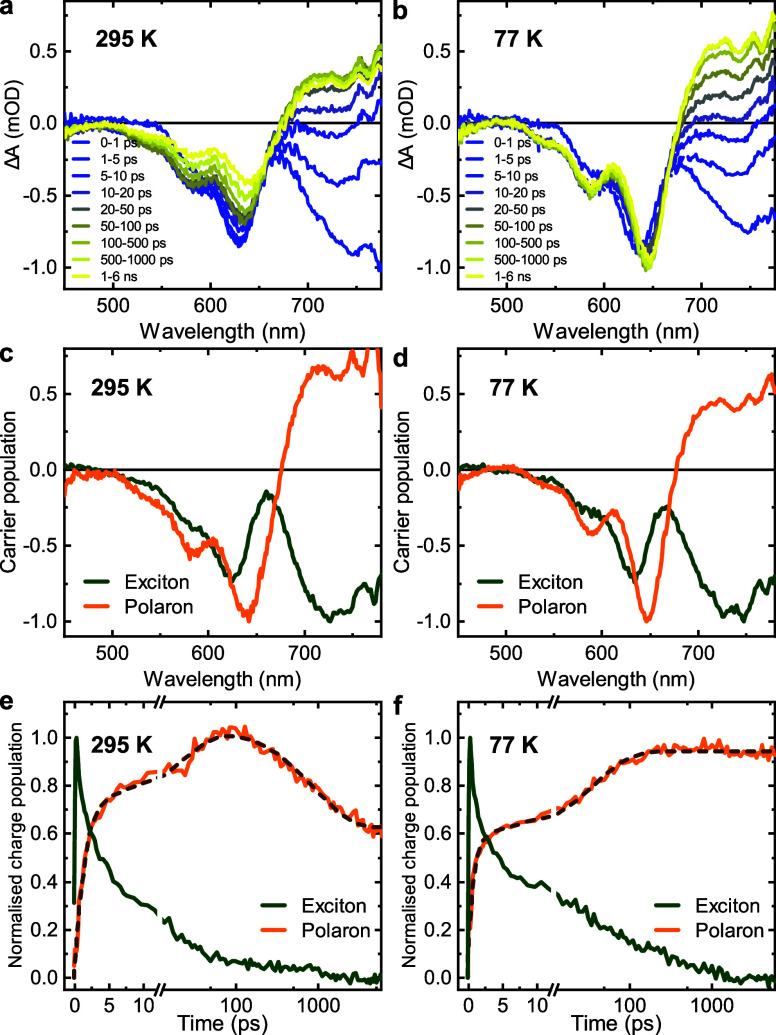
Transient absorption spectroscopic characterization and global analysis (GA) for PBDB-T-2F:BTP-4F blend films at 295 and 77 K. Transient absorption spectra at various pump–probe time delays at (a) 295 K and (b) 77 K. Deconvoluted spectra and the corresponding kinetics for excitons and charges from GA at (c and e) 295 K and (d and f) 77 K. Excitation wavelength was 750 nm, 2.5 μJ cm^–2^.

As an illustrative example, Figures 2a and 2b present the TAS spectra at various time delays for PBDB-T-2F:BTP-4F (PM6:Y6) blend films examined at 295 and 77 K. We note that there have been several recent studies of the detailed photophysics of BTP-4F and PBDB-T-2F:BTP-4F films;^32^ herein, we consider only the temperature dependence of the overall processes of exciton decay and charge generation and recombination. Upon photoexcitation of BTP-4F in the blend at 295 K, the spectrum exhibits two prominent negative bands at 743 and 630 nm, respectively. The band at 630 nm is attributed to the ground state bleach (GSB) of PBDB-T-2F, confirmed by comparison with its ground state absorption, while the band at 743 nm is ascribed to the GSB of BTP-4F in analogy to the spectra of neat BTP-4F (Figure S2). In neat BTP-4F films, this band appears within the instrument response and decays without spectral evolution. However, when BTP-4F is blended with PBDB-T-2F, Figure 2a reveals that the GSB of BTP-4F (at 630 nm) decays to zero within 10 ps, coinciding with the appearance of a broad photoinduced absorption (PIA) signal attributed to the formation of charges (further discussed below). Additionally, it can also be clearly seen that a pronounced shoulder appears at 580–590 nm after 1 ps, indicating hole transfer from BTP-4F excitons to PBDB-T-2F. For time delays >100 ps, the spectra exhibit significant decay, contributing to a 50% reduction in amplitude by 6 ns. Intensity-dependent kinetics probed in this wavelength region (shown in Figure S3) displays a strong dependence of this decay on the excitation intensity, suggesting that the decay results from the bimolecular recombination of charges, which is consistent with that observed in the NIR region (Figure S4). At 77 K, the spectral shapes remain consistent with those observed at 295 K, as shown in Figure 2b, indicating that the same exciton-to-charge conversion processes occur at 77 K. To extract the underlying kinetics of excitons and charges, a genetic algorithm based global analysis was performed on the TAS data from Figures 2a and 2b. The deconvoluted spectra of the excitons and charges at 295 and 77 K are respectively shown in Figures 2c and 2d, along with their corresponding kinetics plotted in Figures 2e and 2f. It can be seen from Figures 2c and 2d that the deconvoluted components exhibit similar spectral shapes at both 295 and 77 K, consistent with the spectral assignments described above. Figures 2e and 2f show that the exciton dissociation rates remain constant as the temperature drops from 295 to 77 K, with a half-life of approximately 4 ps. The kinetics of charge formation, as shown in Figures 2e and 2f, consists of two components wherein the first rise completes within 1 ps while the second rise extends over tens of picoseconds. This slower phase of charge signal rise is assigned exciton diffusion-controlled charge transfer, reported to be particularly pronounced in NFA-based OPVs due to the long exciton diffusion lengths of NFAs.^33,34^ Therefore, for the study herein, we only focus on the fast phase, assigned to charge transfer rate directly at the D:A interface. It is apparent that the charge formation (and exciton decay) kinetics exhibit temperature-independent behavior as indicated by the biexponential fitting in the dotted line. In contrast, it is apparent that the charge decay for delay times >100 ps, observed at 295 K, assigned to bimolecular recombination, becomes negligible at 77 K. In summary, we conclude that for the PBDB-T-2F:BTP-4F blend, while bimolecular recombination is strongly thermally activated, both exciton decay and charge formation exhibit almost temperature-independent kinetics, indicative of activationless charge separation.

In addition to these two temperatures, PBDB-T-2F:BTP-4F films were measured at five intermediate temperatures (Figure S5). Similar GA analyses were carried out for all temperatures to extract exciton and charge kinetics (refer to Figure S6). Extending upon this temperature-dependent TAS characterization and GA analysis, analogous data were also obtained for PBDB-T:EH-IDTBR (Figures S7 and S8), PBDB-T:ITIC (Figures S9 and S14), PBDB-T:PC_71_BM (Figures S10 and S11), PBDB-T-2F:BTP (Figure S12), and PBDB-T-2F:BTP-4F (Figure S13). In each case, optical excitation was of the lowest bandgap component, corresponding to NFA excitation in most blends to drive hole transfer to the donor, and to donor excitation for PBDB-T:PC_71_BM, driving electron transfer to the fullerene acceptor.

**Figure 3:**
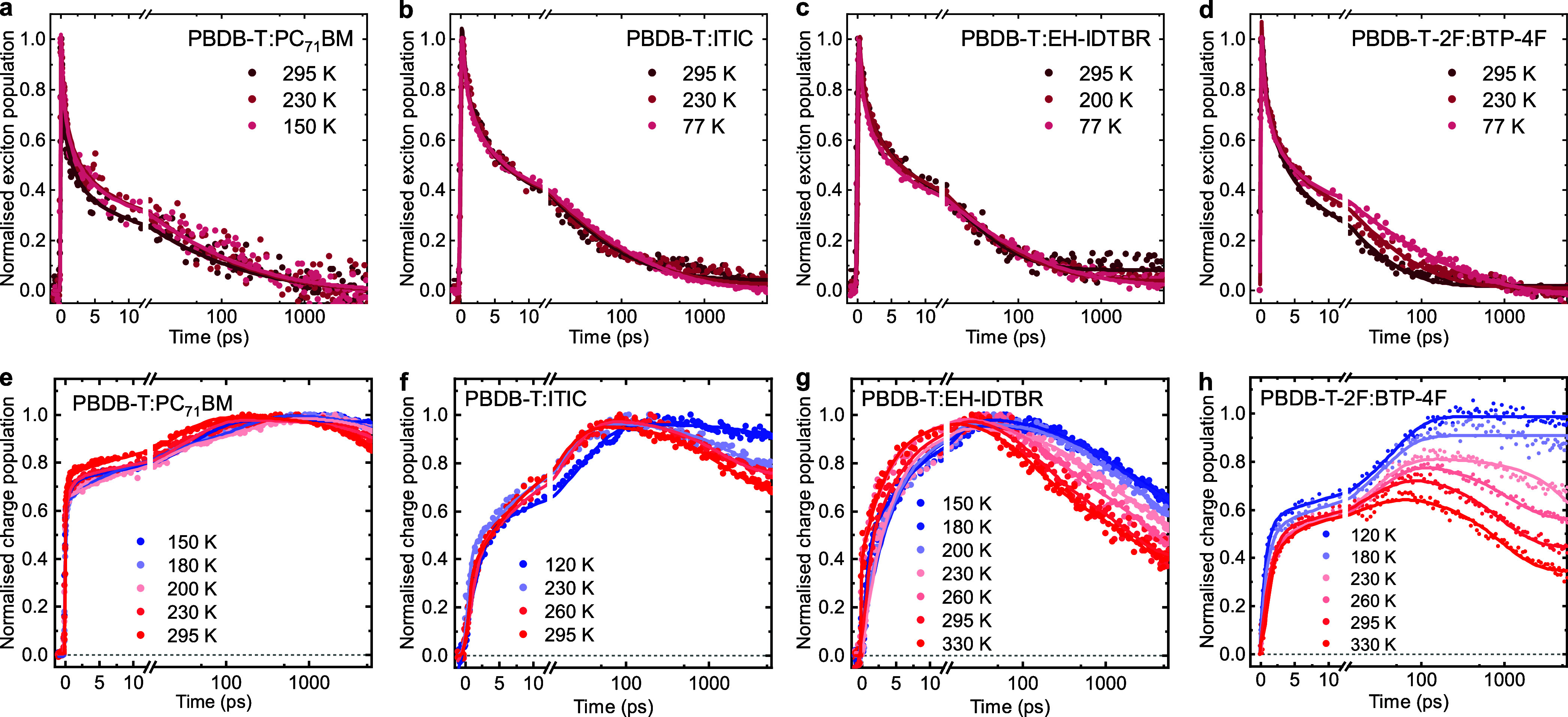
Transient absorption kinetics for excitons and charges at various temperatures for the organic photovoltaic blends including exciton dynamics for (a) PBDB-T:PC_71_BM, (b) PBDB-T:ITIC, (c) PBDB-T:EH-IDTBR, and (d) PBDB-T-2F:BTP-4F and charge dynamics for (e) PBDB-T:PC_71_BM, (f) PBDB-T:ITIC, (g) PBDB-T:EH-IDTBR, and (h) PBDB-T-2F:BTP-4F blend films. Solid lines are multiexponential fittings to the data. Excitation fluences were kept under 5 μJ cm^–2^ to minimize the impact of exciton–exciton annihilation.

Figure 3 shows the resultant exciton and charge kinetics at different temperatures for four of these blends. It is apparent that for all four blends, the exciton decay kinetics and charge rise kinetics exhibit only minor temperature dependence, becoming slightly slower as the temperature is lowered (we note that for PBDB-T:PC_71_BM, the initial charge rise is largely instrument response limited). In contrast, for all four blends the charge decay kinetics, observed for time delays greater than ∼100 ps, exhibit temperature-dependent kinetics. For PBDB-T:PC_71_BM and PBDB-T:ITIC, these decay kinetics are largely slower than our 6 ns time window and so were not analyzed further. For PBDB-T:EH-IDTBR (Figure 3g), the charge decay is fluence-independent (Figure S15) and therefore assigned to the geminate recombination of bound CT states (formally this implies the charges formed are CT states rather than separated charges, however for simplicity we will refer to all such states as charges herein).^30^ It is apparent that the temperature dependence of these geminate recombination kinetics is less pronounced than for the bimolecular recombination kinetics observed for PBDB-T-2F:BTP-4F (Figure 3h). However, the most striking conclusion drawn from these data is that for all four blends studied, the rates of exciton decay and charge formation are largely temperature-independent, as we will further discuss below.

**Figure 4:**
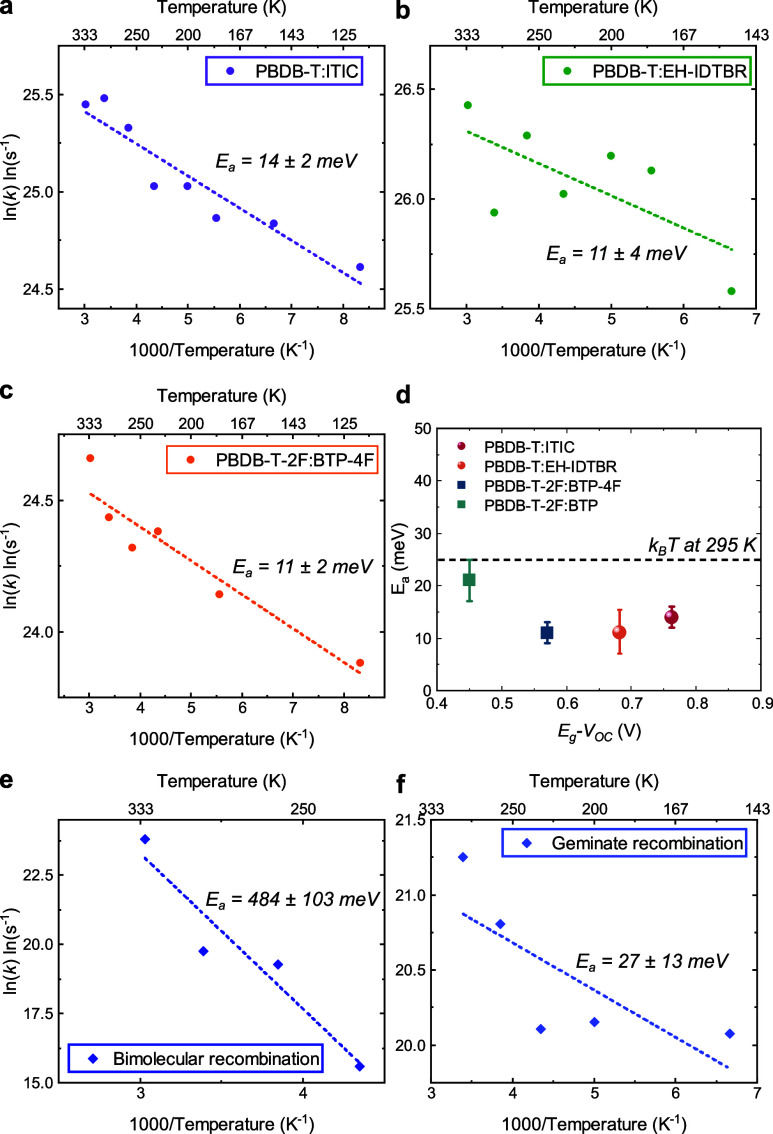
Arrhenius evaluation of the activation energies for polaron generation and recombination in the investigated polymer:NFA blends. Arrhenius plots for polaron generation rates in (a) PBDB-T:ITIC, (b) PBDB-T:EH-IDTBR, and (c) PBDB-T-2F:BTP-4F blends and for (e) bimolecular recombination rates in PBDB-T-2F:BTP-4F and (f) geminate recombination rates in PBDB-T:EH-IDTBR blends. (d) Measured activation energies for polaron generation against the energy loss determined for the corresponding devices *E*_g_ – *V*_OC_, used as a proxy for the energy offset driving charge formation. Uncertainties in our determination of *E*_a_ result primarily from experimental noise.

Arrhenius analyses were employed to determine the activation energies for the charge separation and recombination processes detailed above. These analyses utilized the equation , where *k* represents the rate constant, *E*_a_ is the activation energy, *k*_B_ is the Boltzmann constant, *T* is the temperature, and *A* is the pre-exponential factor. For exciton separation and charge decays, *k* was determined from the first phase of biexponential fits to the data, as discussed previously. For geminate recombination, *k* was obtained from exponential fits to the decay at long times, and for bimolecular recombination, it was derived from decay half-times for power law fits to the data. Figure 4 presents typical Arrhenius analyses, plotting ln(*k*) against the reciprocal temperature, with the slopes yielding the activation energies for the relevant processes (see further discussions in Note S3). First, we focus on the activation energy barriers for charge generation by fitting the charge rise kinetics extracted from Figures 3e–3h into Arrhenius plots for these blends. As shown in Figures 4a–4c, activation energies of 14 ± 2 meV were extracted for PBDB-T:ITIC, and 11 meV were measured for both PBDB-T:EH-IDTBR and PBDB-T-2F:BTP-4F blends. These values are all lower than the thermal energy at room temperature (i.e., ∼25 meV), indicating effectively barrierless and efficient charge generation at room temperature. Fitting the exciton decays yielded similar activation energies. In contrast, the bimolecular charge recombination kinetics observed for PBDB-T-2F:BTP-4F exhibit a much larger activation energy of >400 meV (equivalent to ∼20 *k*_B_*T* at room temperature), as shown in Figure 4e, attributed to thermally activated charge diffusion. Finally, analysis of the CT state geminate recombination observed for PBDB-T:EH-IDTBR yields an activation energy of 27 meV (Figure 4f), most likely due to changes in vibrational wave function overlap as the temperature varies.^35^

Figure 4d plots the measured activation energies for charge formation versus the energy loss *E*_g_ – *V*_OC_ for corresponding devices, where *E*_g_ is the optical bandgap. These results are further summarized in Table S3. *E*_g_ – *V*_OC_ is used as a convenient proxy for Δ*E*_S1-CT_ to circumvent difficulties in measuring CT energies for small energy offset blends. It is apparent that *E*_g_ – *V*_OC_ varies by 300 meV for the blends studied, while *E*_a_ varies by <10 meV. It can thus be concluded that the activation energies for charge generation in the blends studied are essentially independent of *E*_g_ – *V*_OC_ and therefore Δ*E*_S1-CT_. This has important implications for both the reaction mechanism and material energy level optimization, as we discuss further below.

In order to extend further our analysis of the energetic offset dependence of charge generation, analogous TAS data as a function of temperature were obtained for the very low energy offset blend PTO2:BTP-4F (Figure S17). We have previously shown that the very low energy offset in this blend results in substantive hybridization of its exciton and CT states.^36^ While this hybridization complicates quantitative analysis of its charge generation kinetics, the data in Figure S17 clearly indicate that its ultrafast dynamics are essentially temperature-independent, as observed for the other blends studied herein. This result indicates that our observation of activationless charge generation extends even to blends where the energy offset is so small that it results in exciton/CT state hybridization.

Of the blend systems studied herein, the highly efficient low-offset PBDB-T-2F:BTP-4F (i.e., PM6:Y6) blend has been widely investigated by several groups aiming to elucidate the origins of its efficient device performance.^23,37−40^ Our TAS measurements on this blend indicate an activation barrier to charge generation barrier of only ∼11 meV. This is in agreement with the previous reports of effectively activationless charge generation in this blend.^22,23^ Barrierless charge generation has also been previously reported in larger energy offset polymer:fullerene systems. For example, Pensack and Asbury have employed transient vibrational spectroscopy to demonstrate that charge generation in P3HT:PCBM occurs through a barrierless process.^41^ Additionally, Siebbeles et al. showed temperature-independent charge generation yield for the same blend.^42^ However, what is striking from the study herein is that all of the blends studied herein show similarly activationless charge generation. This activationless behavior appears to be independent of the energetic offset driving charge generation, implying a barrierless charge generation pathway regardless of the magnitude of the energetic driving force.

**Figure 5:**
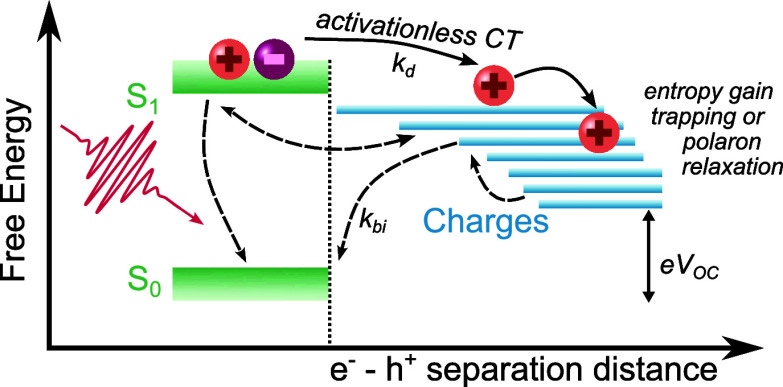
Scheme summarizing the photophysical processes studied herein for blends that achieve efficient charge dissociation without the formation of bound interfacial CT states. Charge dissociation (*k*_d_) is proposed to be adiabatic and activationless. Subsequently, charges are stabilized by polaron formation and charge trapping, resulting in thermally activated bimolecular recombination (*k*_bi_).

Our observation of activationless charge generation independent of energetic offset is at variance with the Marcus nonadiabatic electron transfer theory but is consistent with adiabatic charge transfer models associated with strong coupling between exciton and charge transfer states. This is consistent with previous reports of ballistic charge transfer in PDI:PCBM and PCDTBT:PCBM^43^ and of charge separation in both polymer/fullerene and polymer/NFA blends being mediated via thermally unrelaxed CT states, as illustrated in Figure 5.^43−45^ It has been suggested that such adiabatic charge transfer can be aided not only by larger energy offsets but also by high local carrier mobilities.^46−48^ The dominance of adiabatic charge transfer for the blends studied may therefore at least in part be associated with the delocalized nature of excited states in NFA compared to fullerene acceptors.^35^ Energetic barriers only develop after thermal relaxation of the generated charges, including those associated with polaron formation, which is consistent with our observation of thermally activated bimolecular recombination on longer (>100 ps) time scales.

OPV device performance is often observed to depend strongly on temperature, with the device power conversion efficiency typically dropping at lower temperatures. Our observation of temperature-independent charge generation for several OPV blends indicates such loss of performance is primarily associated with charge extraction rather than charge generation, and in particular from lower carrier mobilities at low temperatures.^49−53^

The study herein is focused on the temperature dependence of charge generation. It includes blends where this charge generation is associated with the generation of separated, free charges which subsequently undergo bimolecular recombination, such as in PBDB-T-2F:BTP-4F. It also includes blends where charge generation results, at least in part, in the generation of interfacial bound CT states, as exemplified by our observation of geminate recombination kinetics in PBDB-T:EH-IDTBR. It is apparent that in both cases, charge generation is essentially activationless. As such, our results indicate that the lower photocurrent densities observed for lower energy offset blends studied herein do not derive from higher activation barriers for charge generation but rather primarily from enhanced geminate recombination losses (e.g., PBDB-T:EH-IDTBR herein)^30^ or from a lower yield of charge transfer due to an unfavorable free energy difference for charge generation (e.g., PBDB-T-2F:BTP and PTO2:BTP-4F herein).^21^ However, our study does not include a consideration of activation barriers for the subsequent separation of any bound CT states. Several studies have reported thermally relaxed CT states exhibiting significant Coulombic binding energy.^30,52−56^ Consequently, dissociation from these thermally relaxed CT states into free charge carriers would be endothermic, resulting in strongly suppressed CT separation into free charges at lower temperatures. However, we also note that other studies have suggested that some systems such interfacial CT exhibit only weak binding energies, enabling efficient CT state dissociation.^57−59^ Such considerations are beyond the scope of this study.

In summary, we have conducted a direct assessment of the activation barriers for charge separation and recombination in a range of fullerene- and nonfullerene-based D/A blend systems using temperature-dependent TAS. TAS measurements reveal that the dynamics of charge generation exhibit negligible temperature dependence, regardless of the magnitude of energy offsets between the D and A. This temperature independence is indicative of charge generation in these OPV blends being activationless, independent of energetic offset. This behavior is indicative of adiabatic rather than nonadiabatic (Marcus) charge transfer, as illustrated in Figure 5. Adiabatic charge generation is consistent with the observation of efficient charge generation in polymer:NFA blends with small energetic offsets and small overall energy losses. The efficiency of charge generation in these blends is most likely enabled by high initial charge delocalization/mobilities, as has been suggested previously for polymer/NFA blends.^60,61^ Charge formation is subsequently stabilized by polaron relaxation/charge trapping, resulting in thermally activated bimolecular recombination. As such, this study not only provides insight into the underlying mechanism of charge generation in OPVs but also suggests how polymer:NFA can achieve efficient charge generation with small energetic offsets and thereby smaller overall energetic loss.

## Supporting Information Available

UV–vis absorption, device *J*–*V* characteristics, electroluminescence (EL), and transient absorption spectroscopy data (PDF)
