# Supplementary material for: Activationless Charge Transfer Drives Photocurrent Generation in Organic Photovoltaic Blends Independent of Energetic Offset
Source: J Am Chem Soc. 2024 Nov 27;146(49):33579–86. doi: 10.1021/jacs.4c11114 (PMC11638955; doi:10.1021/jacs.4c11114)
Supplement: Supplementary file 1 — ja4c11114_si_001.pdf [file ja4c11114_si_001.pdf]

Supplementary Information

For

Activationless Charge Transfer Drives Photocurrent Generation in Organic Photovoltaic Blends Independent of Energetic Offset

Yifan Dong,[1,†] Rui Zheng,[1] Deping Qian,[2] Tack Ho Lee,[3] Helen L. Bristow,[1] Pabitra Shakya Tuladhar,[1] Hyojung Cha,[4,*] James R. Durrant[1,5,*]

[1]Department of Chemistry and Centre for Processable Electronics, Imperial College London, London W12 0BZ, United Kingdom

[2]Straits Institute of Flexible Electronics (SIFE, Future Technologies), Fujian Normal University, Fuzhou, Fujian 350117, China

[3]Department of Chemistry Education, Graduate Department of Chemical Materials, Institute for Plastic Information and Energy Materials, Sustainable Utilization of Photovoltaic Energy Research Center, Pusan National University, 46241 Busan, Republic of Korea

[4]Department of Hydrogen and Renewable Energy, Kyungpook National University, Daegu 41566, Republic of Korea

[5]SPECIFIC and Department of Materials Science and Engineering, Swansea University, Swansea SA1 8EN, United Kingdom

Experimental methods

Sample fabrication: Thin film and device samples were fabricated on glass substrates and ITO substrates. The substrates were cleaned by sonication with detergent, deionised water, acetone and isopropanol, followed by oxygen plasma treatment. For thin films, the oxygen plasma treatment was directly followed by spin-coating the active layer solutions on to the substrates using the same parameters as for devices. For devices, prior to the deposition of the active layer, ZnO layers were deposited first by spin-coating a zinc acetate dihydrate precursor solution (60.4 μL 1-ethanolamine in 2 mL 2-methoxyethanol) followed by annealing at 150 °C for 10 min, giving a ZnO layer of ~30 nm. Details on the processing of active layer solution are detailed below. Following the deposition of the active layers, MoO_3 (10 nm) and Ag (100 nm) layers were deposited by evaporation through a shadow mask yielding active areas of 0.045 cm^2 in each pixel. All the steps apart from depositing the ZnO layer were carried out in a nitrogen glovebox. Specific details for the rest of active layers of film and device fabrication for each material system are summarised below:

PBDB-T:ITIC: an active layer solution of PBDB-T:ITIC (20 mg mL^{-1}, D:A ratio of 1:1) in chlorobenzene/DIO (99.5:0.5 vol%) was spin coated on to the substrates at 2500 rpm for 60 s.

PBDB-T:EH-IDTBR: an active layer solution of PBDB-T:EH-IDTBR (20 mg mL^{-1}, D:A ratio of 1:1) in chlorobenzene was spin coated on to the substrates at 2500 rpm, followed by thermal annealing at 120 °C for 10 mins.

PBDB-T-2F:BTP-4F: an active layer solution of PBDB-T-2F:BTP-4F (18 mg mL^{-1}, D:A ratio of 1:1.2) in chlorobenzene/chloronaphthalene (CN) (99.5:0.5 vol%) was spin coated at 2000 rpm onto the substrates, followed by thermal annealing at 100 °C for 10 mins.

PBDB-T-2F:BTP: an active layer solution of PBDB-T-2F:BTP (18 mg mL^{-1}, D:A ratio of 1:1.2) chlorobenzene/chloronaphthalene (CN) (99.5:0.5 vol%) was spin coated onto the substrates, followed by thermal annealing at 100 °C for 10 mins.

PBDB-T:$PC_{71}BM$: an active layer solution of PBDB:$PC_{71}BM$ (20 mg mL^{-1}, D:A ratio of 1:1.2) in dichlorobenzene:DIO (97:3 vol%) was spin coated onto the substrates, followed by thermal annealing at 100 °C for 10 mins.

PTO2:BTP-4F: an active layer solution of PTO2:BTP-4F (18 mg mL^{-1}, D:A ratio of 1:1.2) with 0.5% additive of CN was spin coated onto the substrates, followed by thermal annealing at 100 °C for 10 mins.[1]

Device current-voltage characterisation: Current-Voltage (J-V) characteristics for devices were measured with a Keithley 2400 source meter ranging from -1.2 V to 1.2 V and were corrected for spectral mismatch. Measurements were carried out under an AM 1.5 G solar illumination of 100 mW cm^{-2} using a filtered 300W xenon lamp (Oriel Instruments) under nitrogen condition. A calibrated silicon photodiode was used as a reference for the J-V measurements.

UV-vis absorption and photoluminescence spectroscopy: Absorption spectra were measured with a spectrophotometer (UV-2600, Shimadzu) with an interval step of 1 nm. Photoluminescence (PL) spectra were measured on a Fluorolog-3 spectrofluorometer (FL 3-22, Horiba Jobin Yvon). Samples were excited at various wavelengths from a Xenon lamp source. The emitted photons were collected in the front face geometry with a photomultiplier tube (PMT) R928P detector with a slit width of 5 nm.

Transient absorption spectroscopy: A commercially available broadband pump-probe femtosecond transient absorption (TA) spectrometer Helios (Spectra Physics, Newport Corp.) was used to measure the TA spectra and kinetics for thin film samples. Ultrafast laser pulses (800 nm, ~100 fs duration) were generated by a 1 kHz Ti:sapphire regenerative amplifier (Solstice, Spectra Physics, Newport Corp.). One portion of the 800 nm pulse was directed to an optical parametric amplifier (TOPAS Prime, Spectra-Physics) and a frequency mixer (Niruvis, Light Conversion) to tune the visible pump pulses into various wavelengths. The pump pulses were modulated at a frequency of 500 Hz by a chopper. The rest of the 800 nm pulse was routed onto a mechanical delay stage with a 6 ns time window and directed through a non-linear crystal (sapphire for the visible region or YAG for the NIR region) to generate a

white light probe ranging from 400-800 or 800-1600 nm. The probe pulse was split into two by a neutral density filter. One portion of the probe pulse served as the reference and was directly sent to the fibre-optic coupled multichannel spectrometers (CCD and InGaAs sensors). The rest of the probe pulse together with the pump pulse were focused onto the same spot on the samples with a beam size of around 0.5 mm^2 before sending it to the spectrometer. Temperature-dependent TAS data was obtained with a liquid nitrogen cryostat (Optistat DN-V, Oxford Instruments) coupled to a temperature controller (MercuryiTC, Oxford Instruments), which allows the tuning of temperatures from 77 K up to 330 K in this work. During temperature-dependent measurements, samples were kept in a vacuum of under 10^{-5} mbar. To compensate the fluctuations, the measured spectrum was normalized to the reference spectrum and averaged for several scans to achieve a good signal-to-noise ratio. Data analysis was performed with the commercialised Surface Xplorer software unless otherwise stated.

Genetic algorithm (GA) analysis: GA analysis was carried out using a previously written programme.[2] Taking the analysis for PBDB-T-2F:BTP-4F as an example, we first extracted the averaged spectra at pump-probe time delays between 0.5-6 ns. As a time-efficient approach, we input the averaged spectra between 0.5-6 ns as initial guesses and add on randomness into each species. Throughout the fitting, the algorithm examines the fitness of each and reaches the most fitted result.

Supplementary Notes

Note S1. Calculation of excitation densities

During transient absorption spectroscopy measurements, low excitation fluences were employed to mimic the charge densities in OPV devices under working condition. Our previous works have shown that typical charge densities in a working OPV device in on the order of ~10^{17} cm^{-3}.[3] Excitation fluence is calculated by dividing the incident excitation energy E by the area of the equipped pinhole (with a radius r), where the excitation energy E is determined by dividing the power P (measured with an energy meter VEGA P/N7Z01560, OPHIR Photonics) against the frequency f of the excitation laser:

$$F\ (J\ cm^{-2}) = \frac{E}{\pi r^2} = \frac{P}{f\pi r^2}$$

The number of incident photons per unit area is then calculated by dividing the incident excitation fluence F by the photon energy E_p at the excitation wavelength λ:

$$n_i\ (cm^{-2}) = \frac{F}{E_p} = \frac{F}{hc/\lambda}$$

The number of photons absorbed by the material (i.e. excitation density) is calculated by considering the sample absorbance A at the excitation wavelength λ and the sample thickness d:

$$n\ (cm^{-3}) = \frac{10^{-A} \times n_i}{d}$$

Note S2. Calculation of activation energies

The Arrhenius equation relates the rate constant of a chemical reaction to the temperature following equation $k = Ae^{-\frac{E_a}{k_B T}}$ where k is the rate constant, E_a is the activation energy of the reaction, k_B is the Boltzmann constant and T is the temperature in Kelvins. In an Arrhenius

plot, $\ln(k)$ is plotted against $\frac{1}{T}$, giving a straight line where the gradient can therefore be used to determine the value for E_a.

Note S3. Discussion on exciton-exciton annihilation

A key consideration in determination of charge transfer kinetics from TAS studies is to confirm that the measured kinetics are not distorted by exciton – exciton annihilation (EEA). The excitation energies employed in our study for the determination of activation energies were under 5 μJ cm^{-2}. Previous literature data, including our own, indicate that EEA kinetics for neat film of the materials employed in our study or in closely analogous materials at this excitation density exhibits time constants of 10-80 ps (see for example Figure S3 in Ref 30, Figure 3 in Ref 44). As discussed in the manuscript, exciton decay and polaron formation exhibited fast (1-5 ps) and slow (10-100 ps) phases, assigned to direct and diffusion limited charge transfer respectively. In this study, our temperature analysis of charge transfer focuses only on the fast phase so as to avoid complications from exciton diffusion. It is apparent that our chosen excitation density is low enough for EEA not to significantly impact this fast phase. Consistent with this conclusion, we note that the neat film exciton decay kinetics, as shown in Figure S2, all exhibit decay times ~ one order of magnitude slower than the fast phase assigned to direct charge transfer in the blends studied.

Supplementary Figures

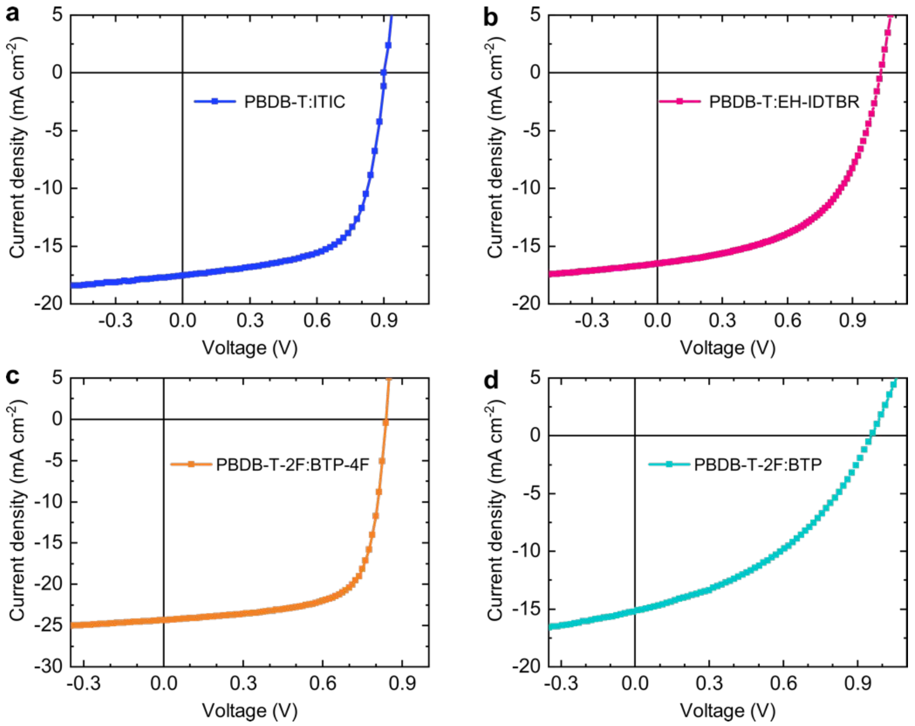

Figure S1. Device photovoltaic performance for the studied blend materials including (a) PBDB-T:ITIC; (b) PBDB-T:EH-IDTBR; (c) PBDB-T-2F:BTP-4F and (d) PBDB-T-2F:BTP.

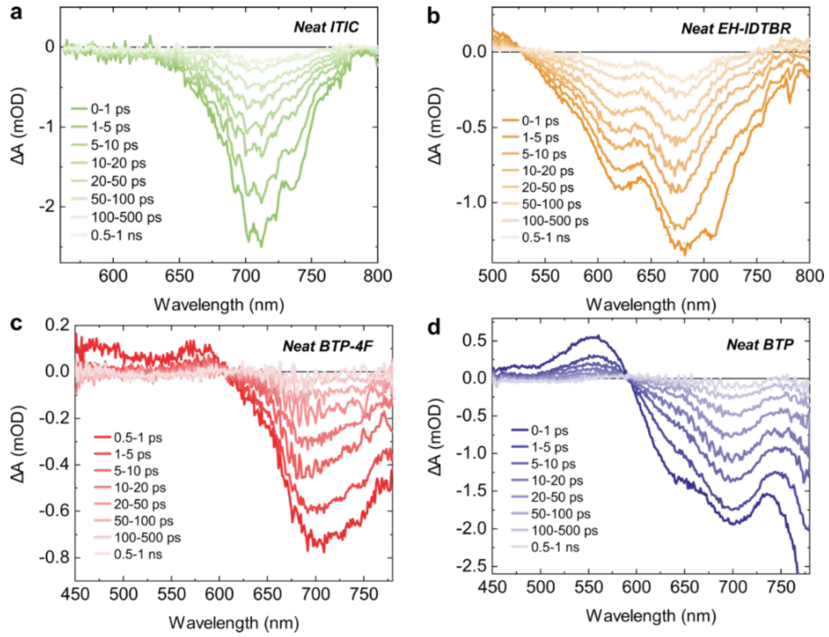

Figure S2. Transient absorption spectra for neat non-fullerene acceptors measured in the visible probe region including: (a) ITIC; (b) EH-IDTBR; (c) BTP-4F and (d) BTP.

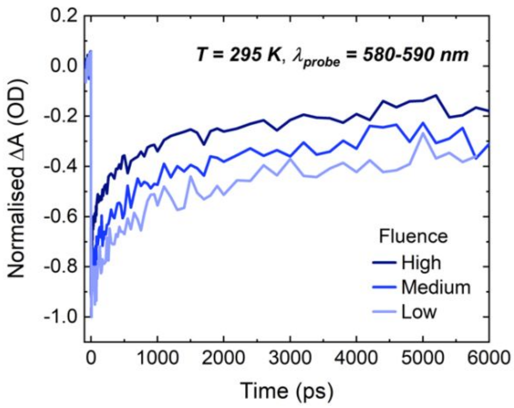

Figure S3. Transient absorption kinetics for PBDB-T-2F:BTP-4F films probed at the donor ground state bleach region, i.e. 580-590 nm. The strong fluence-dependence of the kinetics indicate that the decay is due to bimolecular recombination of polarons.

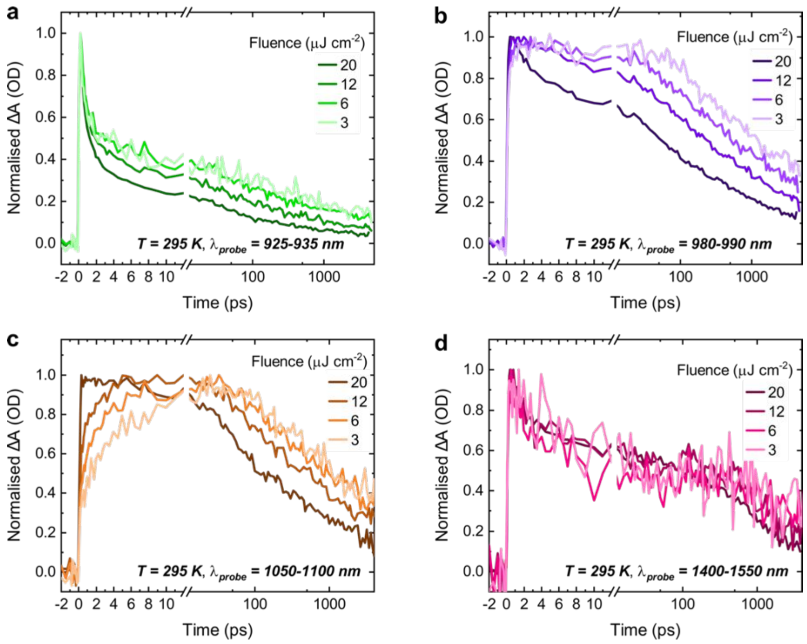

Figure S4. Transient absorption kinetics for PBDB-T-2F:BTP-4F films pumped with various excitation fluences probed and probed at various wavelengths including: (a) 925-935 nm; (b) 980-990 nm; (c) 1050-1100 nm and (d) 1400-1550 nm. The sample was pumped at 750 nm at 295 K. As seen from Figure S13a the PIA signal of BTP-4F excitons at ~925 nm decays within a few ps and red-shifts to form a broader PIA signal centring at ~980 nm, attributed to BTP-4F exciton dissociation. The kinetics in panel (a) clearly shows this sub-ps fast decay phase. At longer pump-probe time delay, the kinetics exhibit a much slower decay as this probe region is overlapped with the charge/polaron PIA signal. Panel (b) shows that the kinetics probed at 980-990 nm exhibit a strong fluence-dependence, indicating this PIA arises from charge/polaron absorption and the fluence-dependent kinetics is a result of the charge bimolecular recombination. While the early-time kinetics at 980-990 nm is strongly overlapped with the exciton PIA, probing at the tail-part (i.e. 1050-1100 nm) of the polaron PIA reveals more information about the hole transfer rate as shown in panel (c). At the lowest excitation fluence of 3 $\mu J\ cm^{-2}$, the hole transfer takes around 40 ps to complete, in agreement with the hole transfer rate obtained from the GA analysis of the TA data in the visible probe (Figure 2e. Panel (d) shows the kinetics probed at 1400-1550 nm which has been assigned to the intermolecular CT exciton between BTP-4F molecules.[4]

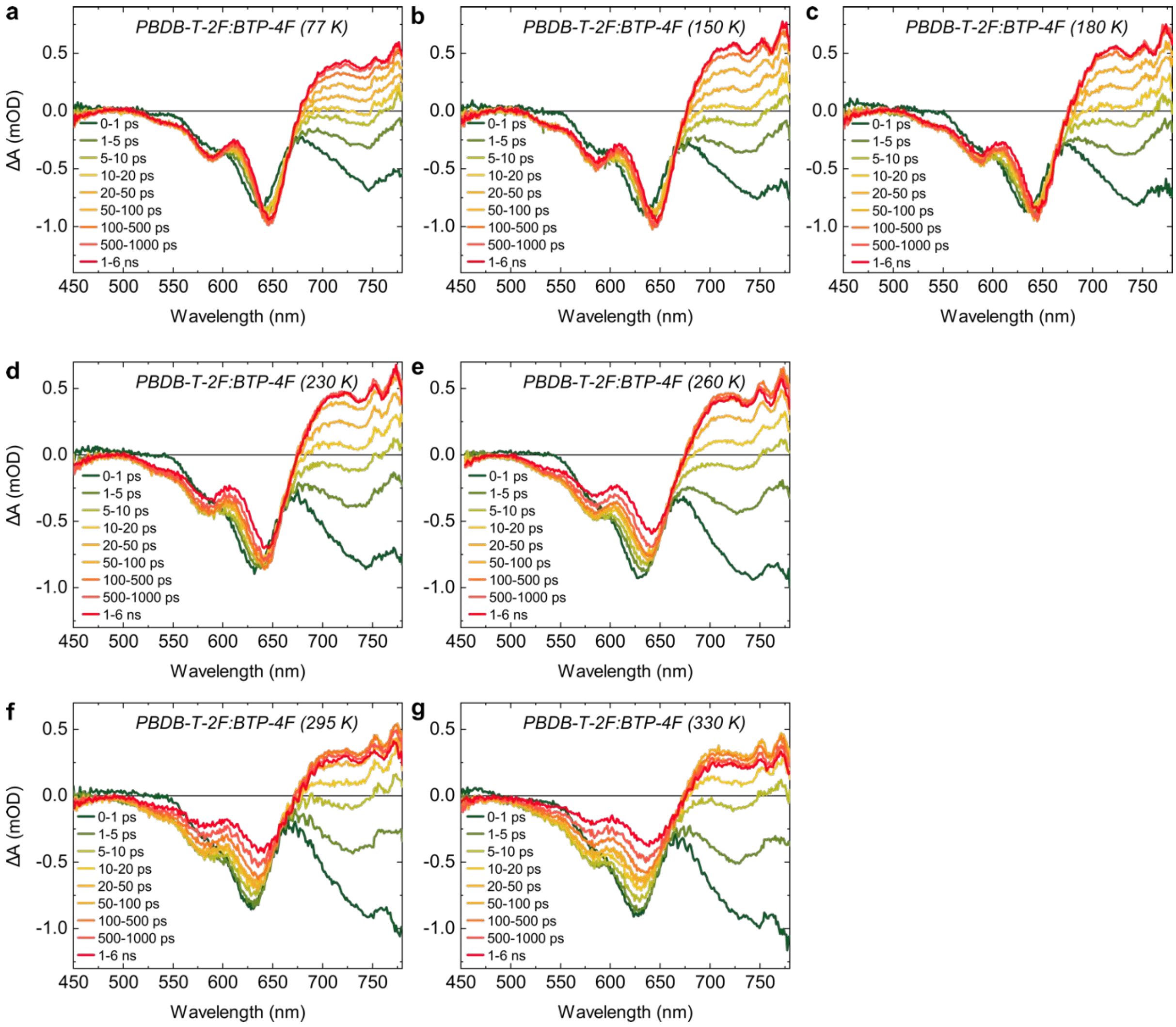

Figure S5. Transient absorption spectra (probed in the visible region) for PBDB-T-2F:BTP-4F films as a function of pump-probe time delay at various temperatures including: (a) 77 K; (b) 150 K; (c) 180 K; (d) 230 K; (e) 260 K; (f) 295 K and (g) 330 K. The sample was pumped at 750 nm with a pump fluence of 4 μJ cm^{-2}. At 0-1 ps after photoexcitation, the spectra are dominated by GSB features with two pronounced bands at 750 nm and 630 nm. The GSB band at 750 nm disappears within 10 ps and is replaced with a broad PIA signal across 673-780 nm, attributed to exciton dissociation into polarons. The decay of this broad polaron PIA signal can only be observed when temperature goes above or equal to 230 K as shown in panels (d)-(g), suggesting the bimolecular recombination gets slowed as temperature decreases.

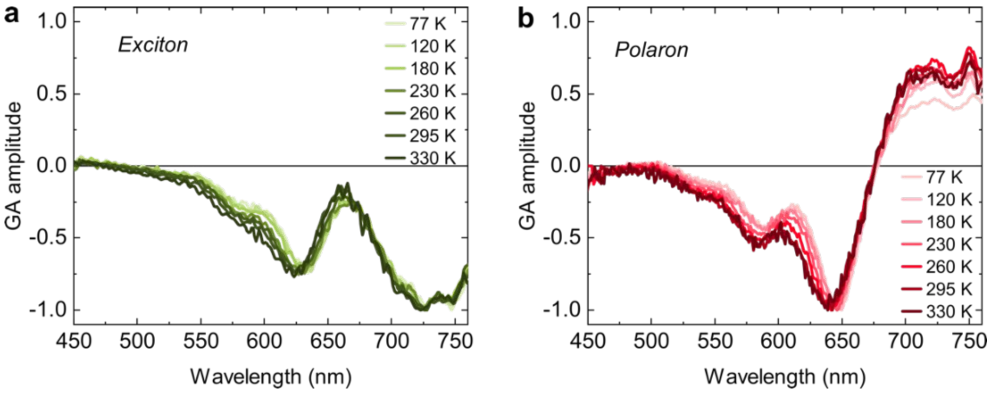

Figure S6. Genetic algorithm (GA) analysis for the transient absorption (TA) data for PBDB-T-2F:BTP-4F films at various temperatures: (a) Normalised exciton spectra at various temperatures decomposed from GA; (b) Normalised polaron spectra at various temperatures decomposed from GA. GA analysis was carried out for TA data at each temperature. As can been seen from the normalised TA spectra, the spectral shape does not exhibit further changes from 500 ps which suggests charge transfer is completed by 100 ps and the TA spectra at time delays > 500 ps is dominated by polarons. Hence, the long-delay-time spectra between 0.5-6 ns was fixed as one of the two components for GA analysis while allowing the evolution of the other component, i.e. excitons. The corresponding kinetics extracted from GA for both excitons and polarons are presented in Figure 2.

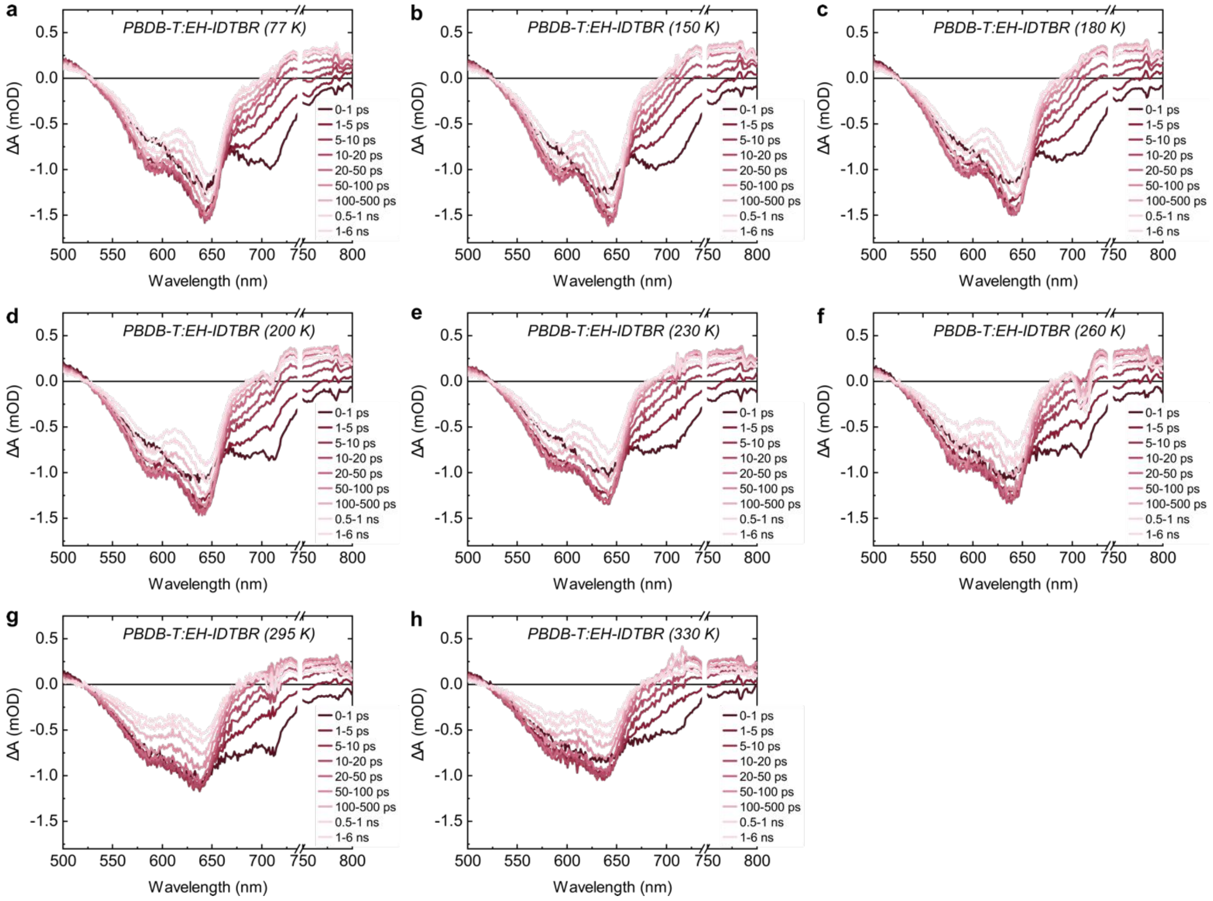

Figure S7. Transient absorption spectra (probed in the visible region) for PBDB-T:EH-IDTBR films as a function of pump-probe time delay at various temperatures including: (a) 77 K; (b) 150 K; (c) 180 K; (d) 200 K (e) 230 K; (f) 260 K; (g) 295 K and (h) 330 K. The sample was pumped at 715 nm with a pump fluence of 3 μJ cm^{-2}. Immediately after photoexcitation, two negative bands situating at 638 nm and ~690 nm emerge. The negative band at ~690 nm is assigned to the GSB of EH-IDTBR by comparing with the TA spectra of neat EH-IDTBR films (shown in Figure S4). As pump-probe time delay increases, a growth in the amplitude of the negative band at 638 nm was observed. In addition, PBDB-T absorbs in the same region (as shown in Figure S1). As such, the appearance of the negative band at 638 nm is attributed to hole transfer from EH-IDTBR to PBDB-T. At time delays > 5-10 ps, an additional band at 580-590 nm emerges and is assigned also assigned to hole transfer from EH-IDTBR exciton to PBDB-T. Hence, the kinetics probed at 580-590 nm reveals the information for hole transfer dynamics while the kinetics probed at 680-690 nm shows the exciton dissociation rate.

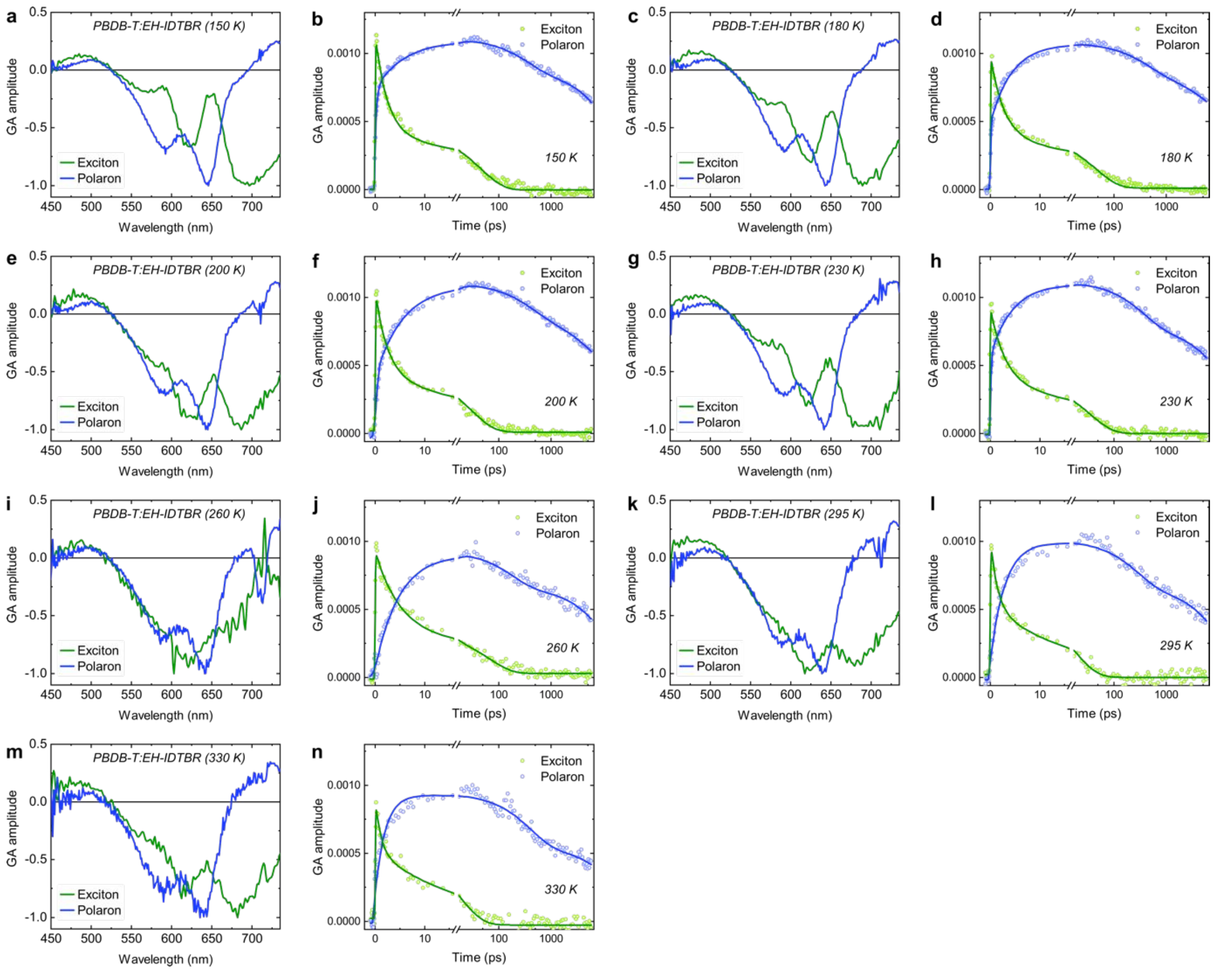

Figure S8. Genetic algorithm (GA) analysis for the transient absorption (TA) data for PBDB-T:EH-IDTBR films decoupled into spectral and kinetic components respectively for excitons and polarons at various temperatures: (a) and (b) 150 K; (c) and (d) 180 K; (e) and (f) 200 K; (g) and (h) 230 K; (i) and (j) 260 K; (k) and (l) 295 K; (m) and (n) 330 K.

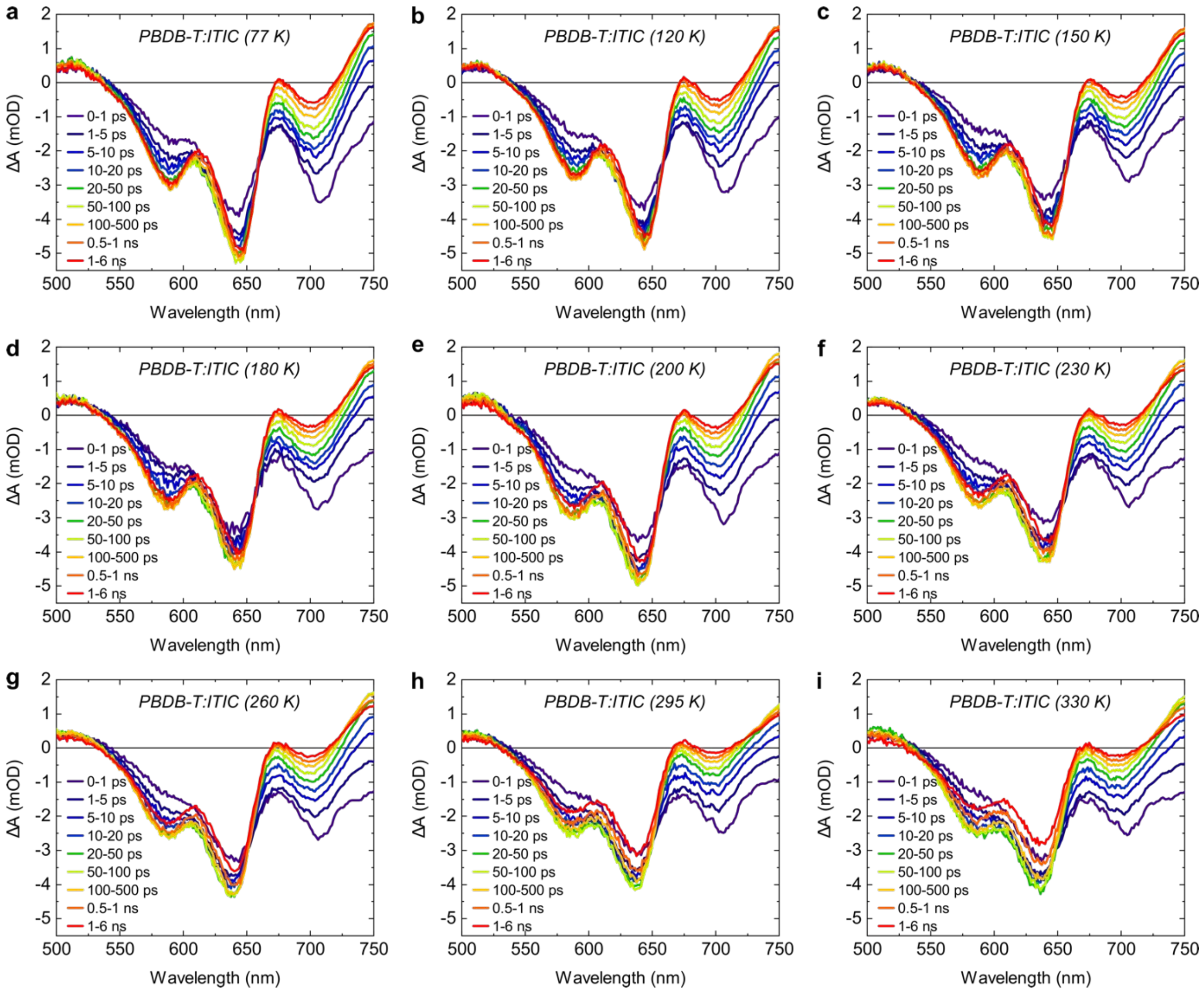

Figure S9. Transient absorption spectra (probed in the visible region) for PBDB-T:ITIC films as a function of pump-probe time delay at various temperatures including: (a) 77 K; (b) 120 K; (c) 150 K; (d) 180 K; (e) 200 K; (f) 230 K; (g) 260 K; (h) 295 K and (i) 330 K. The sample was pumped at 715 nm with a pump fluence of 5 μJ cm^{-2}. Following photoexcitation, two negative bands were observed at 707 nm and 642 nm. The negative band at 707 nm is assigned to the GSB of ITIC excitons by comparing with the TA spectra of neat ITIC (as shown in Figure S2). The negative band at 642 nm arises from the GSB of the donor PBDB-T, which then exhibits a slow rise over tens of ps and then a moderate decay. The rise of the GSB signal at 642 nm is due to the hole transfer from ITIC to PBDB-T while the decay of this feature is due to the recombination of polarons. In addition to these two negative bands, another negative band situating at 588 nm emerges as pump-probe time delay increases, which is also attributed to the hole transfer from ITIC to PBDB-T.

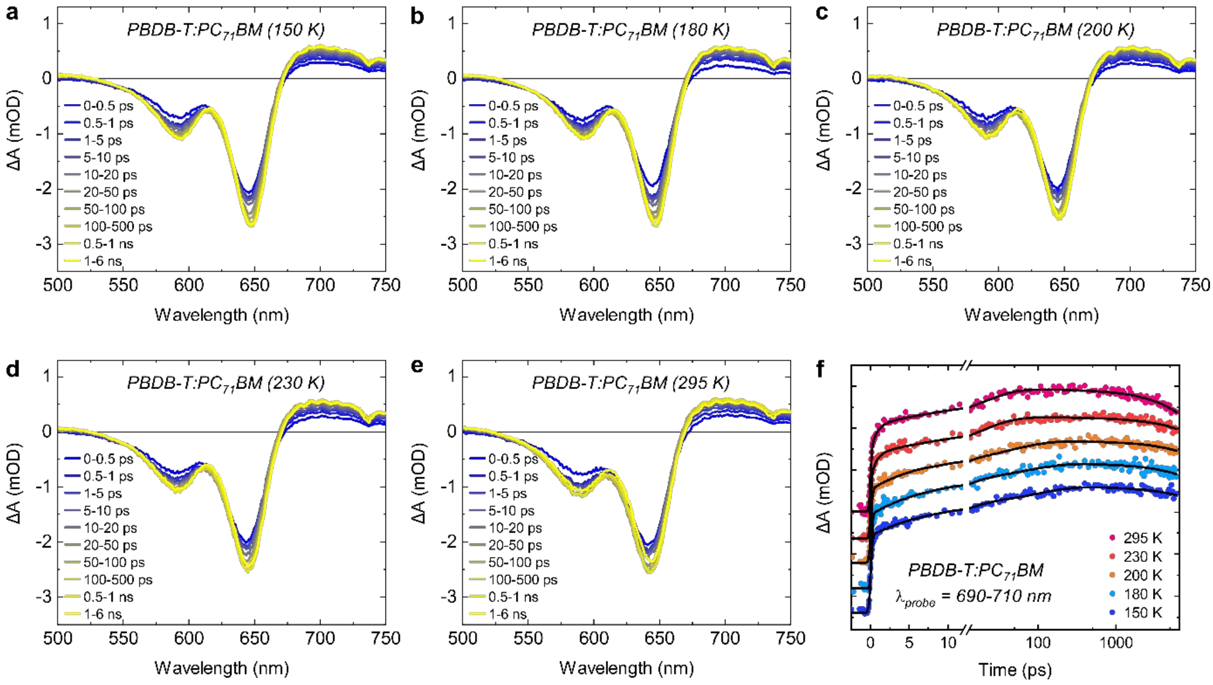

Figure S10. Transient absorption characterisation (in the visible region) for PBDB-T:$PC_{71}BM$ films: transient absorption spectra as a function of pump-probe time delay at various temperatures including: (a) 150 K; (b) 180 K; (c) 200 K; (d) 230 K and (e) 295 K; (f) normalised transient absorption kinetics probed at 690-710 nm (assigned to the photoinduced absorption of polarons) where each kinetics traces are vertically shifted in order to show better comparison among these traces.

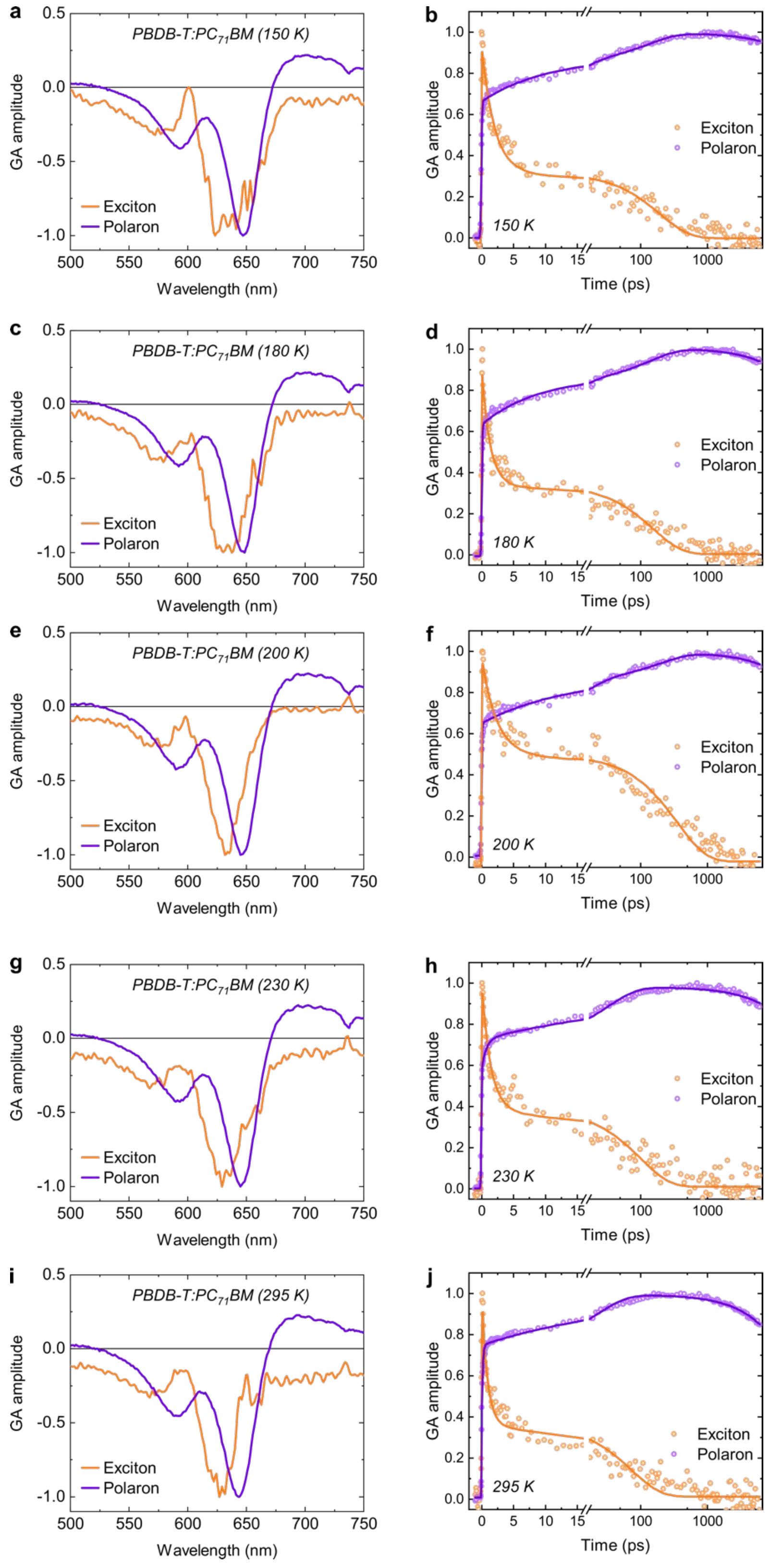

Figure S11. Genetic algorithm (GA) analysis for the transient absorption (TA) data for PBDB-T:$PC_{71}BM$ films decoupled into spectral and kinetic components respectively for excitons and polarons at various temperatures: (a) and (b) 150 K; (c) and (d) 180 K; (e) and (f) 200 K; (g) and (h) 230 K; (i) and (j) 295 K.

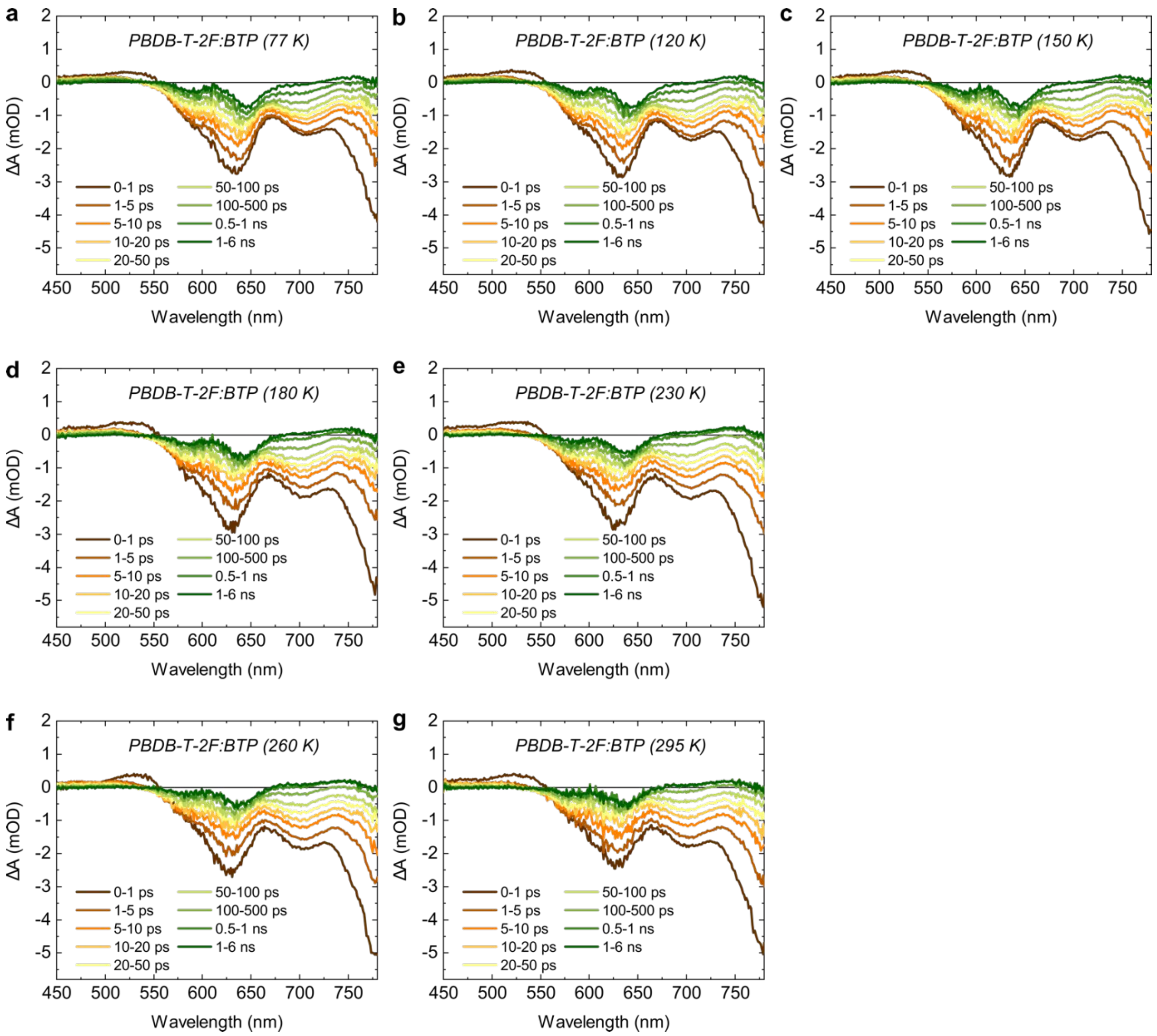

Figure S12. Transient absorption spectra (probed in the visible region) for PBDB-T-2F:BTP films as a function of pump-probe time delay at various temperatures including: (a) 77 K; (b) 120 K; (c) 150 K; (d) 180 K; (e) 230 K; (f) 260 K and (g) 295 K. The sample was pumped at 750 nm with a pump fluence of 10 μJ cm^{-2}.

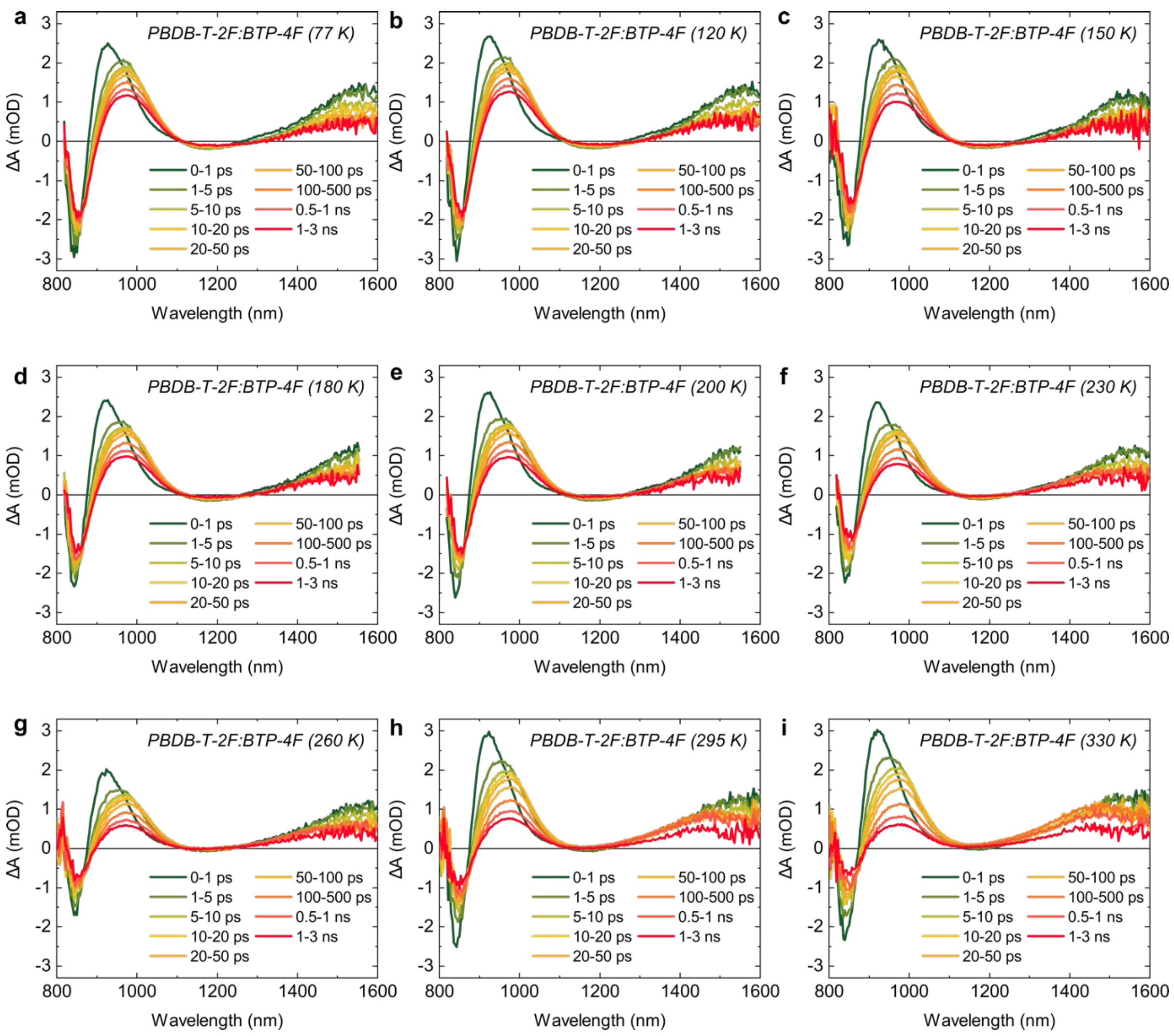

Figure S13. Transient absorption spectra (probed in the near-infrared region) for PBDB-T-2F:BTP-4F films as a function of pump-probe time delay at various temperatures including: (a) 77 K; (b) 120 K; (c) 150 K; (d) 180 K; (e) 200 K; (f) 230 K; (g) 260 K; (h) 295 K and (i) 330 K. The sample was pumped at 750 nm with a pump fluence of 6 μJ cm^{-2}. Immediately following photoexcitation, a strong PIA signal at 925 nm appears and this has been previously assigned to BTP-4F singlet exciton absorption by us and several other groups.[6–8] This PIA signal rapidly red-shifts to the lower-energy region and peaks at ~980 nm within a few ps, attributed to exciton dissociation to polarons. The broad PIA signal at ~980 nm is absent in neat BTP-4F films, hence it is assigned to polarons (see further discussions in Figure Sx). In addition to the PIA signal at 925 nm, other features including a GSB signal at 850 nm as well as another PIA signal centring at 1500 nm can also be observed.

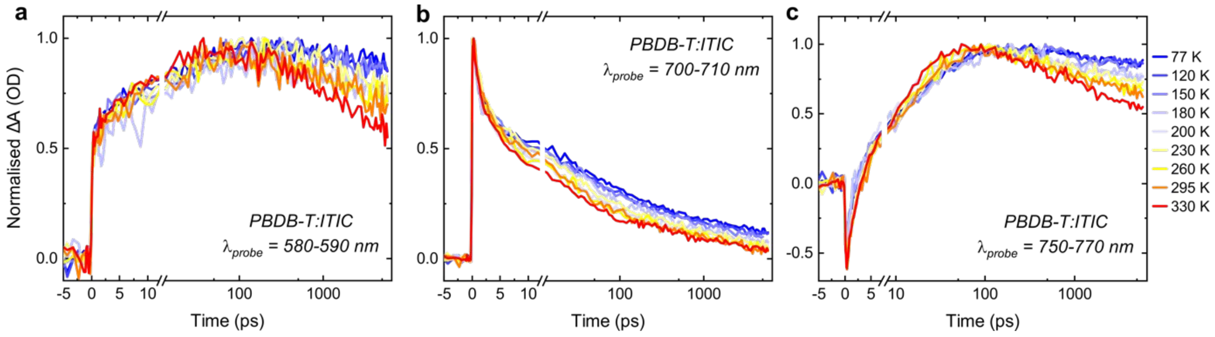

Figure S14. Normalised transient absorption kinetics for PBDB-T:ITIC films at various temperatures including 77 K, 120 K, 150 K, 180 K, 200 K, 230 K, 260 K, 295 K and 330 K with probe wavelengths at: (a) 580-590 nm; (b) 700-710 nm and (c) 750-770 nm. The sample was pumped at 715 nm with a pump fluence of 5 μJ cm^{-2}.

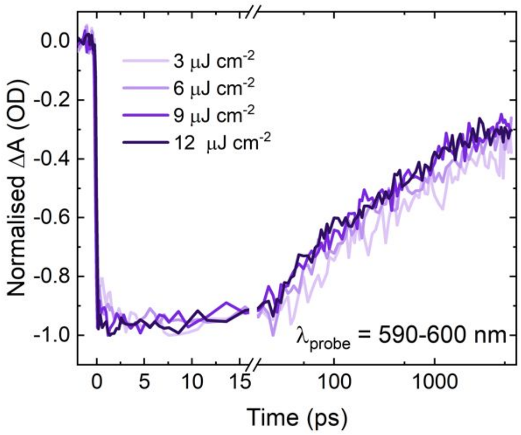

Figure S15. Transient absorption kinetics measured at various excitation fluences for PBDB-T:EH-IDTBR films probed at 590-600 nm.

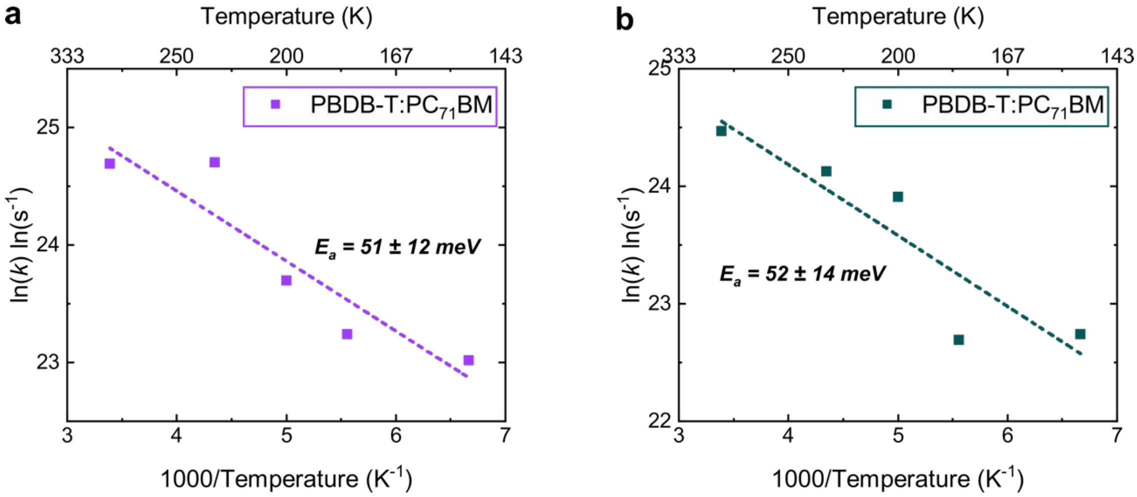

Figure S16. Comparing Arrhenius plots for PBDB-T:PC$_{71}$BM films. (a) the kinetic rates were extracted by fitting the rises at 690-710 nm in the raw transient absorption data shown in Figure

S10f; (b) the kinetic rates were extracted from the polaron dynamics shown in Figure S11 following genetic algorithm analysis.

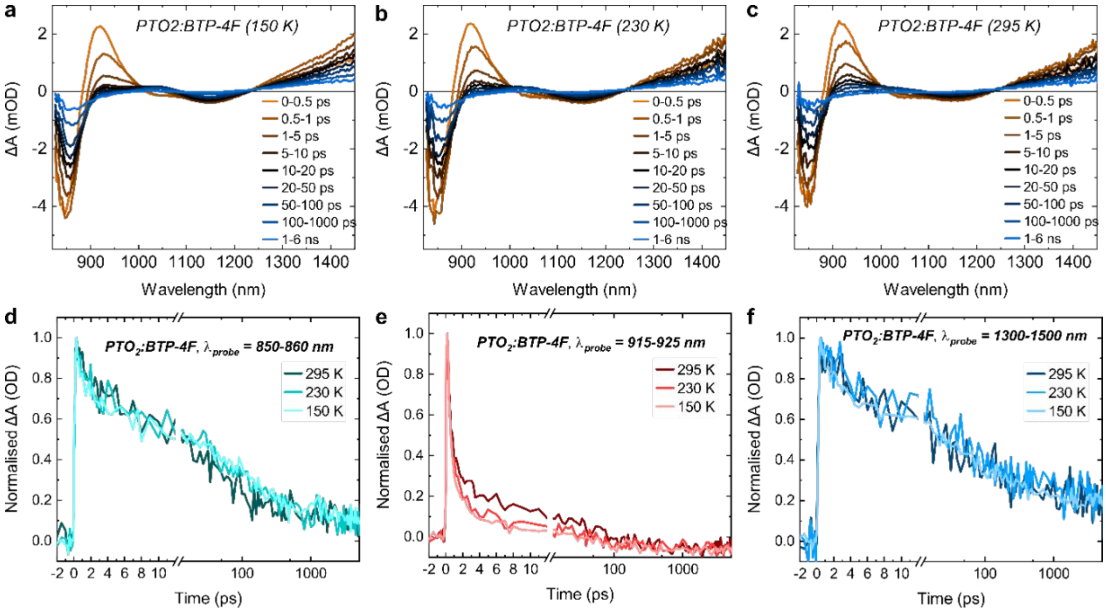

Figure S17. Transient absorption characterisation (probed in the near-infrared region) for PTO2:BTP-4F films at various temperatures: TA spectra at (a) 150 K; (b) 230 K, (c) 295 K and TA kinetics probed at (d) 850-860 nm, (e) 915-925 nm, (f) 1300-1500 nm. The sample was pumped at 750 nm with a pump fluence of 8 μJ cm^{-2}. The initial PIA signal at 0-1 ps following photoexcitation is due to the BTP-4F singlet excitons as previously assigned. As pump-probe time delay increases, this PIA signal decays. As temperature decreases to 295 K and 150 K, an additional broad PIA signal between 1000-1050 nm emerges.

Supplementary Tables

Table S1. Photovoltaic parameters for the organic photovoltaic blends studied in this work.

Materials	J_{SC} (mA cm^{-2})	V_{OC} (V)	FF (%)	PCE (%)
PBDB-T:$PC_{71}BM$				
PBDB-T:EH-IDTBR	14.96	1.000	58.0	8.66
PBDB-T:ITIC	16.80	0.900	74.0	11.2
PBDB-T-2F:BTP	15.20	0.957	40.8	5.92
PBDB-T-2F:BTP-4F	24.35	0.838	70.0	14.3
PTO2:BTP-4F	21.70	0.918	51.2	10.2

Table S2. Fitting parameters for the polaron kinetics in PBDB-T-2F:BTP-4F films by fitting the rise with two exponents with $y = A_1 \times \exp(x/t_1) + A_2 \times \exp(x/t_2) + y_0$.

Temperature (K)	τ_1 (ps)	τ_2 (ps)
77	0.74 ± 0.1	39.4 ± 1.6
150	0.82 ± 0.1	42.6 ± 2.0
180	0.86 ± 0.1	32.8 ± 2.7
230	1.10 ± 0.1	25.8 ± 2.0
260	1.22 ± 0.07	27.8 ± 1.7
295	1.55 ± 0.10	24.5 ± 4.1
330	1.84 ± 0.18	19.6 ± 3.2

Table S3. Table summarizing the 295 K rate constant, the activation energy and the $E_g/q-V_{OC}$ energy gap.

Materials	Rate constant at 295 K (ps^{-1})	Activation Energy (meV)	$E_g/q-V_{OC}$ (V)
PBDB-T:EH-IDTBR	2.60 ± 0.2 ps	11 ± 4	0.68
PBDB-T:ITIC	1.20 ± 0.2 ps	14 ± 2	0.76
PBDB-T-2F:BTP	1.84 ± 0.1 ps	21 ± 4	0.45
PBDB-T-2F:BTP-4F	0.17 ± 0.02 ps	11 ± 2	0.57

Given the huge discrepancies in the reported energetics values, we used $E_g/q-Voc$ values instead. The correlation between Voc vs energetics can be found in our previous work.[5]

Supplementary References

1. D. Qian, S. M. Pratik, Q. Liu, Y. Dong, R. Zhang, J. Yu, N. Gasparini, J. Wu, T. Zhang, V. Coropceanu, X. Guo, M. Zhang, J.-L. Bredas, F. Gao, J.R. Durrant, *Adv. Energy Mater.* 2023, 13, 2301026.

2. S. Gélinas, O. Paré-Labrosse, C. N. Brosseau, S. Albert-Seifried, C. R. McNeill,, K.R. Kirov, I. A. Howard, R. Leonelli, R. H. Friend and C. Silva, *J. Phys. Chem. C*, 2011, **115**, 7114-7119

3. J. Wu, J. Luke, H. K. H. Lee, P. Shakya Tuladhar, H. Cha, S.-Y. Jang, W. C. Tsoi, M. Heeney, h. Kang, K. Lee, T. Kirchartz, J.-S. Kim and J. R. Durrant, *Nat. Commun.,* 2019, **10**, 5159

4. R. Wang, C. Zhang, Q. Li, Z. Zhang, X. Wang and M. Xiao, *J. Am. Chem. Soc.*, 2020, **142**, 12751-1275

5. D. Credgington, J. R. Durrant, *J. Phys. Chem. Lett.* 2012, **3**, 1465-1478.
